# Functional analysis of *ZmMADS1*a reveals its role in regulating starch biosynthesis in maize endosperm

**DOI:** 10.1038/s41598-019-39612-5

**Published:** 2019-03-01

**Authors:** Qing Dong, Fang Wang, Jingjing Kong, Qianqian Xu, Tingchun Li, Long Chen, Hongjian Chen, Haiyang Jiang, Cheng Li, Beijiu Cheng

**Affiliations:** 1 Maize Research Center, Anhui Academy of Agricultural Sciences, Hefei, 230031 China; 2 National Engineering Laboratory of Crop Stress Resistence, Anhui Agricultural University, Hefei, 230036 China

## Abstract

MADS-box family proteins play an important role in grain formation and flower development; however, the molecular mechanisms by which transcription factors regulate the starch metabolism pathway are unclear in maize. Here, we report a transcription factor, *ZmMADS1a*, that controls starch biosynthesis in maize (*Zea mays* L.). We demonstrate the expression of *ZmMADS1a* in tassel, silk, and endosperm, and show that the protein is localized to the cell nucleus. Compared with the control, seeds of overexpressing *ZmMADS1a* increased starch content (especially amylose content), had smaller starch granules and altered chemical structure. Meanwhile, overexpression of *ZmMADS1a* resulted in increases in the contents of soluble sugars and reducing sugars in maize. *ZmMADS1a* plays a positive regulatory role in the starch biosynthesis pathway by up-regulating several starch biosynthesis related genes. We also show that *ZmMADS1a* has a similar adjustment mechanism of starch biosynthesis in rice. Collectively, our study suggests that *ZmMADS1a* functions as a positive regulator of starch biosynthesis by regulating the expression of key starch metabolism genes during seed development.

## Introduction

Based on domain structure, rates of evolution, developmental function, and the degree of functional redundancy, the MADS-box transcription factor family can be divided into two types of MADS-box proteins; Type I (the SRF superfamily) and Type II (the MEF2 superfamily), which contain three additional sequence domains and differ in their DNA binding properties compared to Type I^1^. The biological function of Type I proteins, which are weakly expressed in all species’ tissues, may participate in male and male gametophyte development and early development of the embryo and endosperm^2–4^; Four *Arabidopsis* of Type I genes have been cloned, including *AGL23*, *AGL28*, *AGL61*, and *AGL62*, which play important roles in embryonic development, flowering time, female gamete differentiation, and formation of the endosperm cells, respectively^5–8^. Studies describing the biological function of Type II genes are fairly common, and these proteins are known to participate in different flowering regulation pathways which promote or delay flowering time by regulating the expression of genes involved in flower morphological differentiation, and they also participate in the regulation of fruit and seed development^9^. Type II MADS-box domain usually have only one exon^10^ and, similar to the bZIP transcription factors, can form dimers^11^. Dimers of MADS-box transcription factors can bind to CArG-boxes, conserved DNA elements with the consensus sequence 5′-CC[A/T]_6_GG-3′^12^, which are recognized by the MADS-box domain.

Functional studies in maize and rice have shown that Type II MADS-box genes play important roles in the processes of grain formation and flower development. In maize, the *bearded-ear* gene can regulate the normal development of the corn flower^13^, the *ZmMADS1* and *ZmMADS3* genes affect the growth of the maize ear^14^, and *ZmMADS2* and *ZmMADS4* regulate the development of the inflorescence and the formation of pollen, respectively^15^. Loss of the ZmMADS19 protein leads to significantly larger glumes and androgynous corn^16^; the *ZmMADS47* gene can regulate maize prolamine directly to affect endosperm storage activity by interactions with *Opaque2*^17^. Type II MADS-box genes have also been well studied in rice. *OsMADS57* plays a negative regulatory role in regulating the expression of *DWARF14* (*D14*) through the interaction of miR444 with OsTb1 in rice shoots^18^; RNAi silencing of the *OsMADS29* gene can lead to the severe *shrunken* seed phenotype, and this regulatory factor can also bind targets directly involved in programmed cell death (PCD)^19^.

In our study, we identified and characterized the *ZmMAD1a* gene, which encodes a protein that belongs to the MIKC^C^-group of TypeII. MADS-box proteins. MIKC^c^-group genes control various aspects of sporophyte development^20,21^. The MIKC^c^-group genes are classified by phylogenetic analysis into 12 major subfamilies that include AG, AGL2, AGL6, AGL12, AGL15, AGL17, AP3/PI, GGM13, STMADS11, TM3, AP1/SQUA, and *FLOWERING LOCUS C* (*FLC*). Expression of some MIKC^C^-group genes from rice, tomato, and *A*. *thaliana*, among others, is affected by stress treatment, indicating that they are involved in regulating flowering time in response to stress^22^. For instance, the *AGL12* gene affects flowering time in *A*. *thaliana*^23^, and *GmNMH7* is expressed in different periods of flower development in soybean, and expression in root nodules was also found to be controlled by photoperiod^24^. The *OsMADS29* gene is associated with the degradation of the nucellus and nucellus swelling in rice^19^. Some genes in the MIKC^c^-group have been cloned such as *ZAG1*, *ZAG*2, *ZAG3*-*ZAG5*, and *ZMM3*-*ZMM8*^25^. *ZAG3* is expressed in the inflorescence and can affect the growth of the meristem in maize^13^. These genes are especially prominent on almost all levels of the gene regulatory network that controls reproductive development in flowering plants, but seed growth regulation has not been extensively studied.

In the present study, we identified and characterized the *ZmMAD1a* gene, which encodes a MADS-box transcription factor. Overexpression of the *ZmMAD1a* gene resulted in changes to the structures and contents of starch, and changes in sugar content, as well as changes in the expression levels of starch biosynthesis genes in maize and rice. Our study will clarify the mechanism underlying the molecular regulation of starch synthesis by *ZmMAD1a*, and will lay the foundation for further analyses of the starch metabolic pathways in maize.

## Results

### ZmMADS1a structural characteristics and gene expression analyses

**Figure 1:**
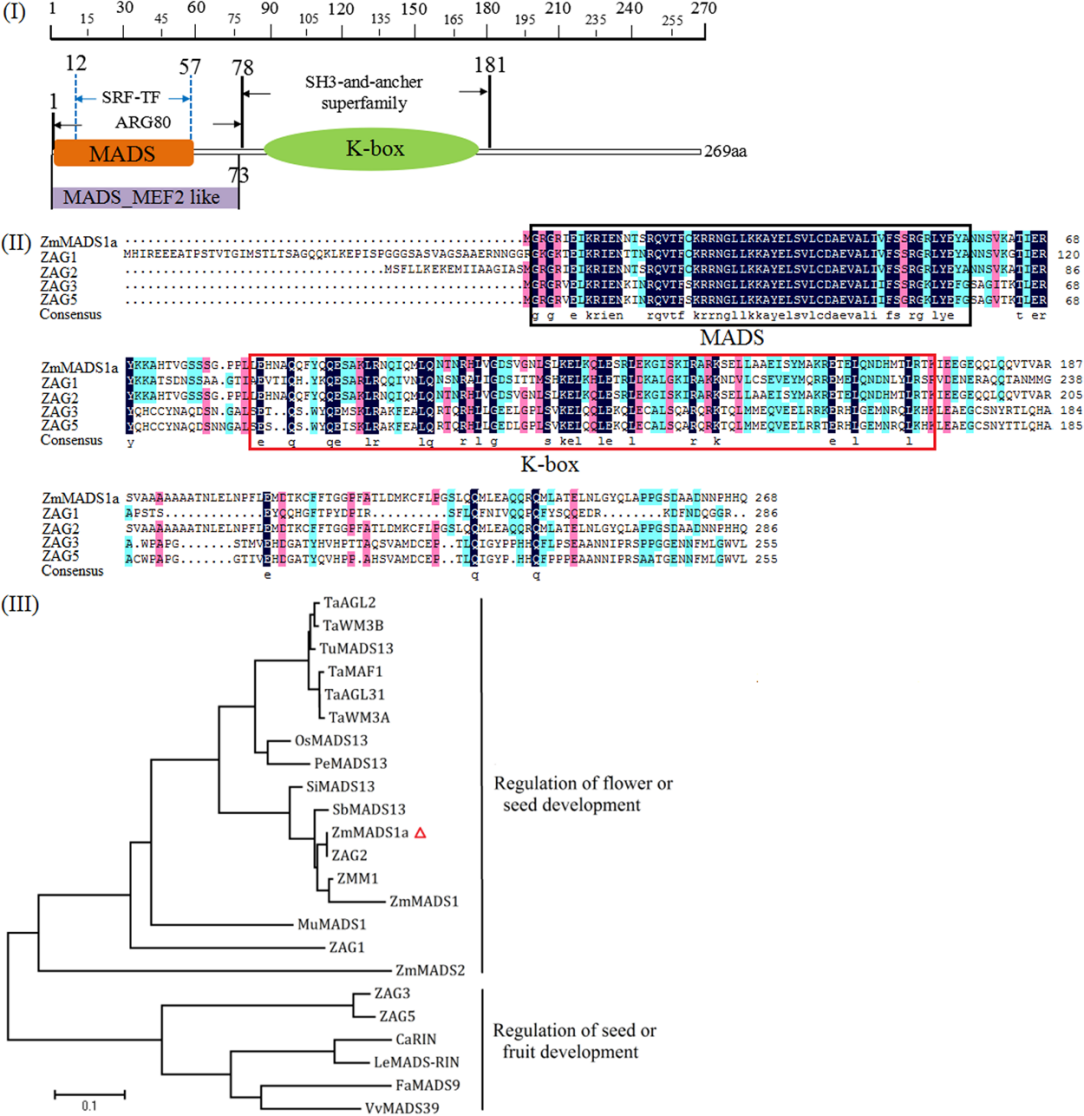
Protein sequence analysis and alignment of ZmMADS1a with other maize MADS-box proteins. (I) Protein structure analysis of ZmMADS1a. Sequence domains are MADS (2–58 aa), MCM1, Agamous, Deficiens, and SRF; MADS_MEF2_like (2–73 aa), MEF2-like/Type II subfamily of MADS box family of eukaryotic transcriptional regulators; K-box (84–172aa), which is commonly found associated with SRF-type transcription factors. (II) Homology analysis of amino acid sequences. ZmMADS1a was aligned with ZAG1, GRMZM2G052890_P01; ZAG2 GRMZM2G160687_P03; ZAG3, GRMZM2G160565_P01; ZAG5, GRMZM2G003514_P01. Different colors represent different conservativeness. The black box denotes the MADS box, the red box represents K-box. (III) Phylogenetic analysis based on an alignment of the ZmMADS1a sequence with MADS-box proteins from other plant species. The ZmMADS1a is indicated by the red triangle. The accession numbers are listed as follows: TaAGL2, ABF57921; TaWM3B, CAM59043; TuMADS13, EMS56192; TaMAF1, AHA51692; TaAGL31, AHA49652; TaWM3A, CAM59042; OsMADS13, AF151693_1; PeMADS13, AHM92069; SiMADS13, XP_004960786; SbMADS13, XP_002441989.1; ZAG2, XP_020405641; ZMM1, CAA57073; ZmMADS1, AQK40454; MuMADS1, DQ060444; ZAG1, NP_001105321.1; ZmMADS2, AAO85643;ZAG3, NP_001105332.1; ZAG5, NP_001105692.2; CaRIN, DQ999998; LeMADS-RIN, AF448522_1; FaMADS9, AF484683; VvMADS39, XM_002263003.

**Figure 2:**
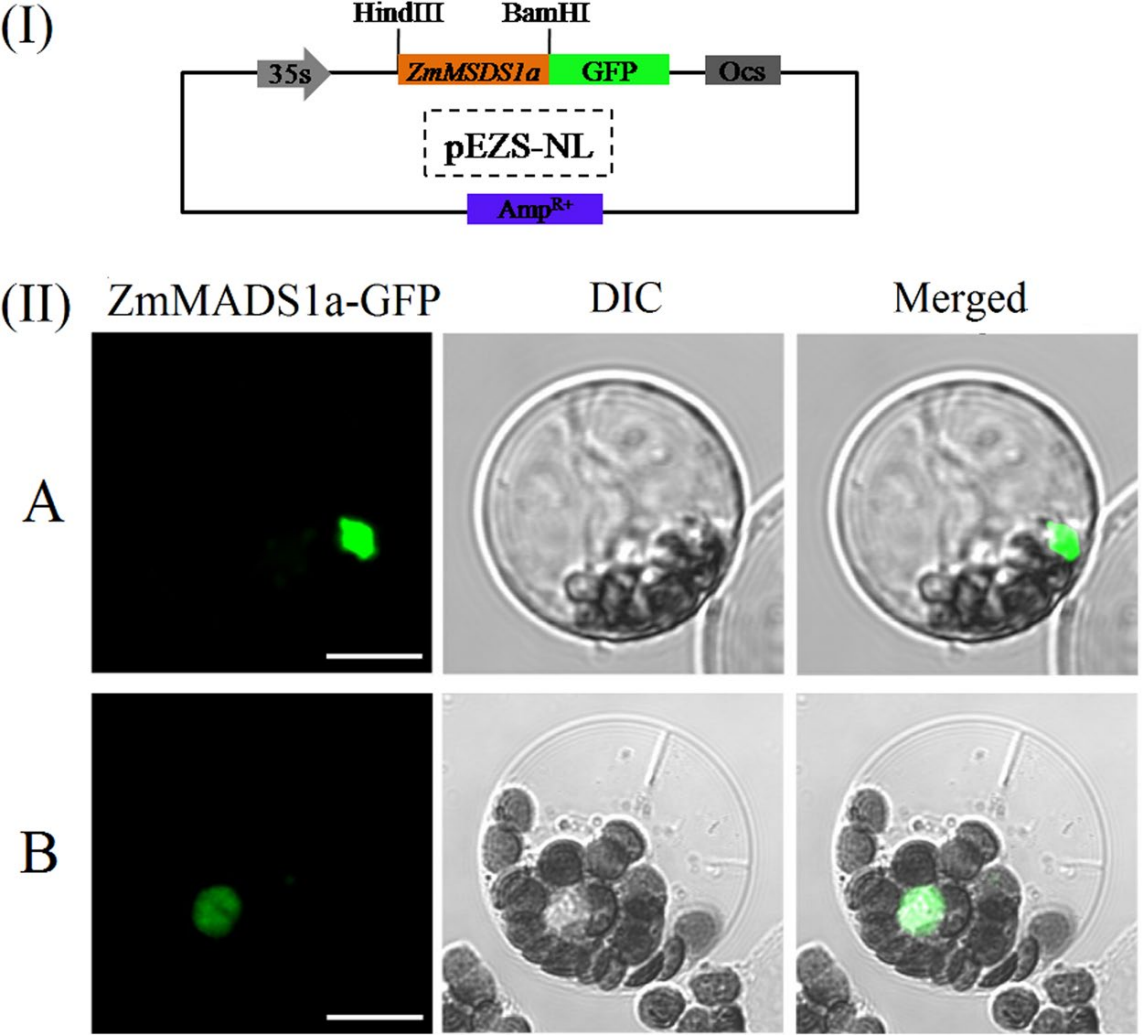
Subcellular localization of the *ZmMADS1a* protein. (I) Simplified map of the pEZS-pBI-Zm*MADS1a*-GFP construct; (II) the ZmMADS1a protein is localized to the cell nucleus; (**A**) Arabidopsis (**B**), maize; Scale bar = 20 μm.

ZmMADS1a is predicted to encode a polypeptide of 269 amino acids with a calculated molecular weight of 30.23 kDa (Fig. 1I). Like other MADS-box family proteins, ZmMADS1a contains the MADS box (2–58aa) and K box (84–172aa) domains at the N-terminus that are key to the functions of MADS-box proteins. Sequence comparisons indicate that *ZmMADS1a* codes for a putative Type II MIKC^C^ transcription factor. The protein also has a MADS_MEF2 like (Myocyte Enhance Factor 2-like) domain which can bind DNA and exist as hetero- and homo-dimers, and has an important effect in homeotic regulation in plants. The protein also has an ARG80 region which is a regulator of arginine metabolism and related MADS-box-containing transcription factors (Fig. 1I). The deduced amino acid sequences were used in BLAST searches and the best hits, four AG-like genes from *Zea mays* (*ZAG1*, *ZAG2*, *ZAG3*, *ZAG5*), are highly conserved with respect to their MADS and K domains (Fig. 1II). Phylogenetic analysis indicates that ZmMADS1a is closely related to ZAG2 (*zag2*), ZmMADS1 and other proteins that function in the regulation of flower and seed development (Fig. 1III). The functional properties of ZmMADS1a were analysed, and the results show that ZmMADS1a is localized to the cell nucleus (Fig. 2).

**Figure 3:**
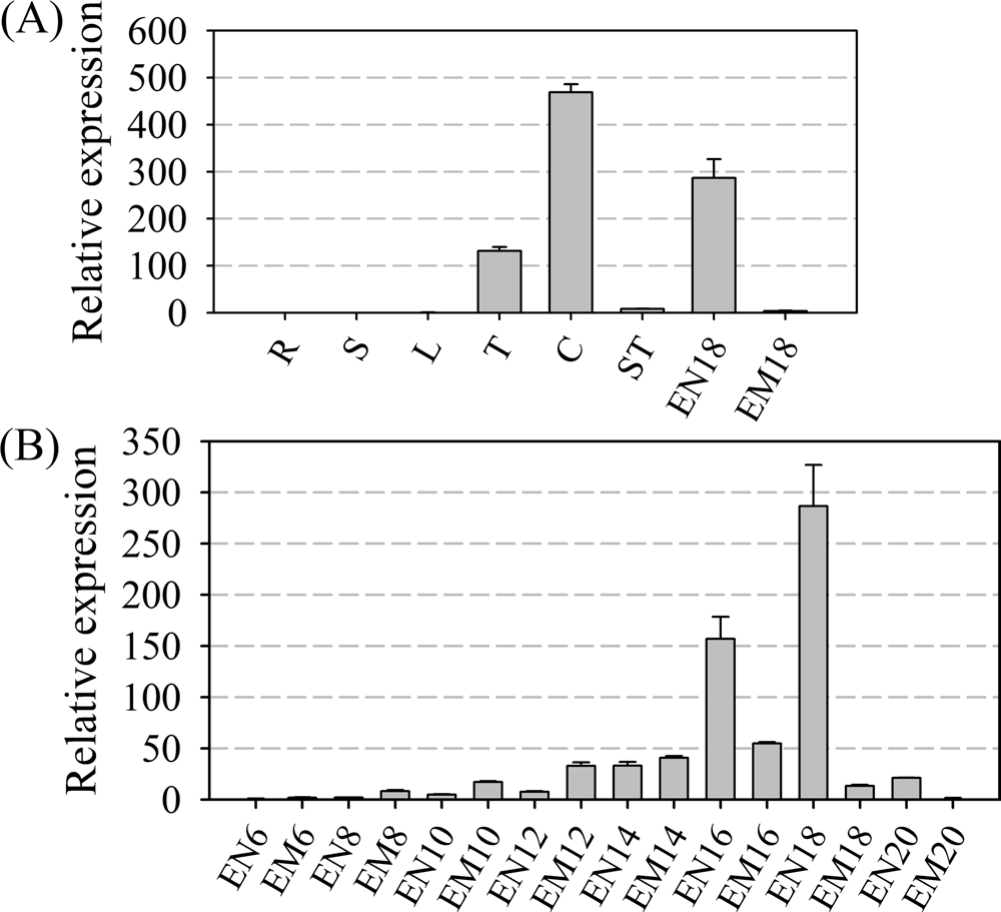
Expression analysis of *ZmMADS1a* in maize by qRT-PCR. (**A**) Expression of *ZmMADS1a* in eight different maize tissues (**B**) Expression of *ZmMADS1a* in embryo (Em) and endosperm (En) at eight stages of seed development. R, root; S, stem; L, leaf; T, tassel; C, comsilk, S, stegophyll; En6, 6-DAP endosperm; Em6, 6-DAP embryo; En8, 8-DAP endosperm; Em8, 8-DAP embryo; En10, 10-DAP endosperm; Em10, 10-DAP embryo; En12, 12-DAP endosperm; Em12, 12-DAP embryo; En14, 14-DAP endosperm; Em14, 14-DAP embryo; En16, 16-DAP endosperm; Em16, 16-DAP embryo; En18, 18-DAP endosperm; Em18, 18-DAP embryo; En20, 20-DAP endosperm; Em20, 20-DAP embryo. All data are presented as mean ± SD from three replicates. Relative expression was calculated by setting the expression of *ZmMADS1a* in the stem as 1.0.

To understand the expression pattern of *ZmMADS1a*, we performed quantitative qPCR using RNA extracted from tissues at different developmental stages. *ZmMADS1a* was found to be expressed at a relatively high level in the tassel, silk, and endosperm (Fig. 3A). We sampled embryos and endosperm every two days from 6- to 20-DAP (days after pollination). We found that the relative expression of *ZmMADS1a* increased gradually in the endosperm until 14-DAP, after which it increased by 3-fold at 16-DAP, peaked at 18-DAP, and then decreased to approximately the 10-DAP level in 20-DAP endosperm (Fig. 3B).

### Overexpression of *ZmMADS1a* regulates starch metabolism genes

**Figure 4:**
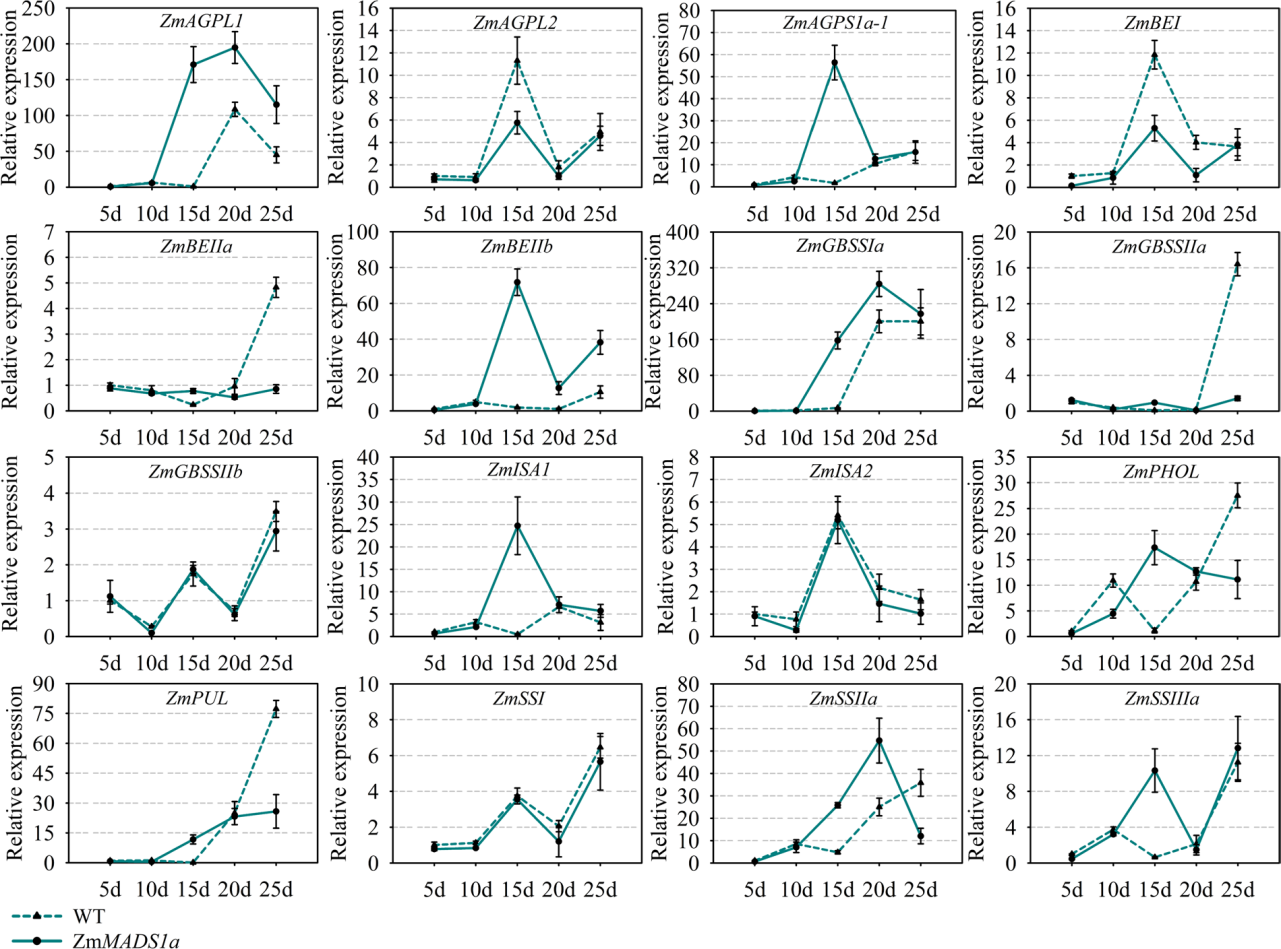
Expression of 16 maize starch biosynthesis genes during endosperm development in wild-type and the *ZmMADS1a* overexpressing lines. The expression level of each gene in the 5 DAP endosperm of ‘ZZC01’ maize seeds was used as the control. Values represent the mean ± SD of three replicates.

To investigate the function of *ZmMADS1a* at the whole plant level, we generated a transformation vector containing the full-length *ZmMADS1a* sequence driven by the CaMV 35S promoter (*Pro*_*35S*_*:ZmMADS1a*), and transformed the maize line ‘ZZC01’. Sixteen starch biosynthesis genes were selected for analysis of gene expression levels in the maize endosperm between the transgenic *ZmMADS1a*-overexpressing lines and the wild-type at different days after pollination. Transcription of *ZmAGPL1*, *ZmAGPS1a*, *ZmBEIIb*, *ZmGBSSIa* (*wx*), *ZmISA1*, and *ZmSSIIa* showed significant increases in the T_3_-generation transgenic lines. The expression levels of *ZmAGPL2*, *ZmBEI*, and *ZmBEIIa* genes were lower than the wild-type in the whole stage. The gene *ZmAGPL2* and *ZmBEI* were down-regulated at 15d. The expression of *ZmPUL* and *ZmGBSSIIa* was low at 20 DAP but increased rapidly at 25 DAP, while *ZmGBSSIIb*, *ZmISA2* and *ZmSSI* showed almost no change in expression over the course of the experiment compared to wild-type (Fig. 4). We also transformed the *Pro*_*35S*_*:ZmMADS1a* construct into the *japonica* rice cultivar ‘Zhonghua 11’. Transcription of *OsAGPS2b*, *OsAGPS1*, *OsBEIIb*, *OsISA1*, and *OsISA2* was significantly increased in the T_3_-generation transgenic lines. The expression level of *OsGBSSIa* (*wx*) increased rapidly after 15 DAP, peaked at 20 DAP, and then fell to the control level by 25 DAP. The expression levels of *OsAGPL2*, *OsBEI* and *OsSSIIIa* were significantly down-regulated compared to the control. *OsAGPL1* and *OsSSI* showed almost no change in relative expression from 3 to 25 DAP compared to the wild-type control (Fig. S1). Comparing the gene expression levels *of ZmMADS1a* in maize and rice, we found that many of the gene homologs (*BEIIb*, *ISA1*, *SSIIa*, *GBSSIa*, *AGPL2*, *BEI*, *PUL*, and *SSI*) had similar expression patterns, and have an important influence on the regulation of starch biosynthesis in both species. This indicates that the *ZmMADS1a* gene is extensively involved in the regulation of starch and glucose metabolism in maize endosperm.

### Ectopic expression of *ZmMADSla* results in grain morphology

Nineteen maize T_3_ lines were randomly selected for the analysis of grain morphology. Panicle length, panicle width, panicle circumference, and 100-seed weight showed no obvious changes in *ZmMADSla*-overexpressing transgenic maize plants compared to the wild-type ‘ZZC01’ (Fig. S2). We further field tested the homozygous T_3_ rice lines and compared them with wild type (‘Zhonghua 11’) and the empty vector (1301a) transgenic plants. Seeds of all of the *ZmMADSla* transfomants were slightly longer than the controls (Fig. S3I,III), but there were no significant changes in the 1000-seed weights (Fig. S3II). We also compared other phenotypes in the maize and rice T_3_ families. In maize, both plant height and ear height were reduced compared to wild-type plants, but leaf length and leaf width were not significantly different (Tables S4, S5). These results indicate that overexpression of the *ZmMADSla* gene has no significant effects on grain morphology.

### Effects of ectopic expression of *ZmMADS1a* on starch granule size and structure

**Figure 5:**
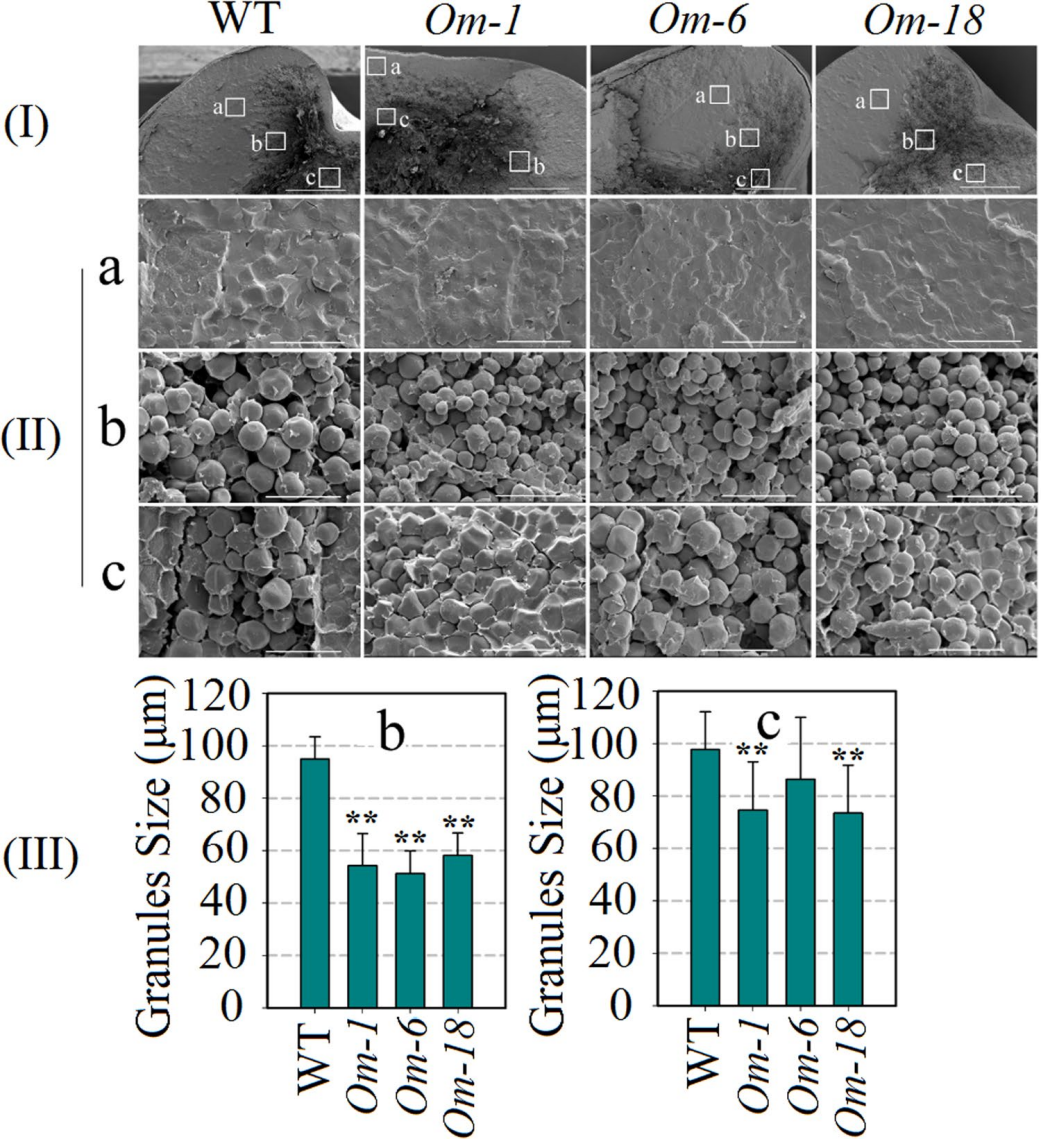
Comparisons of starch granule size and structure in maize endosperm. WT, wild-type plants (‘ZZC01’); *Om-1*, *Om*-6, and *Om*-18 are three *ZmMADS1a*-overexpressing maize lines; a-c, position of endosperm; (I), Bar = 1 mm; (II), Bar = 30 μm; (III) Starch granules size of b and c. To investigate the possible effects of *ZmMADS1a* overexpression on starch granules in the endosperm, transverse sections of seeds from T_3_-generation plants were observed by scanning electron microscopy (SEM). Three regions were compared in the maize seed sections. No changes were observed in the first region due to the compacted granules (**a**). There were obvious changes in the second regions in the three lines, with the granules from the transgenic seeds being smaller than in wild-type (**b**). The third areas of the seeds from the three lines showed only slight changes in granule size compared to wild-type (**c**). The asterisks indicate that the correlation coefficients were highly significantly different (*P < 0.05, **P < 0.01).Values represent the mean ± SD of triplicates.

To investigate the possible effects of *ZmMADS1a* overexpression on starch granules in the endosperm, transverse sections of seeds were observed using scanning electron microscopy (SEM). Three regions were compared in the maize seed sections (Fig. 5). No changes were observed in the first region due to the compacted starch granules (Fig. 5a). The second region from the three lines showed noticeable changes in granule size, with the granules from the transgenic seeds being smaller than in the wild-type (Fig. 5IIb,IIIb), while granules from the third region of the endosperm were only slightly smaller than in wild-type (Fig. 5IIc,IIIc).

The ^13^C {^1^H} CP/MAS spectra for starch granules isolated from rice and maize endosperm are shown in Fig. S4. Signals from 94–105 ppm were assigned to C1, signals from 68–78 ppm were assigned to C2, C3, and C5, signals at 58–65 ppm were associated with C6, and the signal at 82.0 was assigned to C4. The chemical shifts of each major peak are presented in Tables S7, S9. From Fig. S4I we can see that all of the chemical shifts in the *ZmMADS1a*-overexpressing maize lines are moved to the left about 0.5 ppm compared with wild-type. To calculate peak area in maize, the peak area from 52.1–112 was taken as 100%, and was also divided into four parts (C1, 112–88.5 ppm; C4, 88.5–78.4 ppm; C2, C3, C5, 78.4–65.1 ppm; C6, 65.1–52.1 ppm). Compared with the wild-type, the peak areas of C1 and C4 increased slightly, the area of C6 decreased slightly, and the area of the C2, C3, C5 peak was almost unchanged in the *ZmMADS1a*-overexpressing maize lines (Table S8). The variation in peak areas showed similar results in rice (Fig. S4II, Table S10). These results indicate that overexpression of the *ZmMADS1a* gene can change the chemical structure of starch in both maize and rice.

### Overexpression of *ZmMADS1a* improves starch and sugar contents

**Figure 6:**
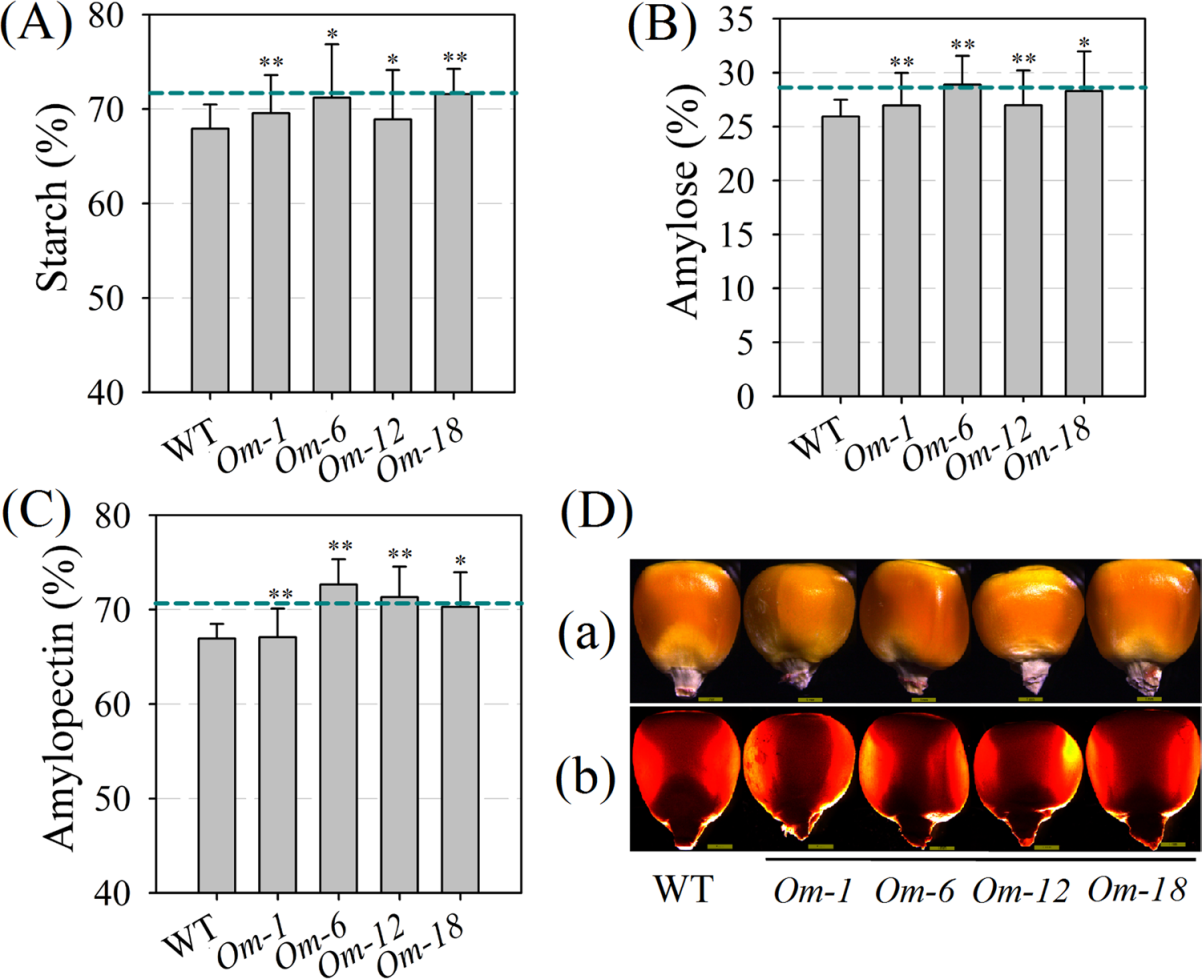
Endosperm starch content in seeds of *ZmMADSla-*overexpressing transgenic maize lines and wild-type. (**A**) total starch content; (**B**) amylose content; (**C**) amylopectin content; (**D**) light transmission; *Om-1*, *Om-6*, *Om-12*, and *Om-18* are four overexpressing lines of *ZmMADSla* seeds; WT, wild-type plants (‘ZZC01’). The dashed blue lines indicate the average for the overexpressing lines. The asterisks indicate that the correlation coefficients were highly significantly different (*P < 0.05, **P < 0.01).Values represent the mean ± SD of triplicates.

**Figure 7:**
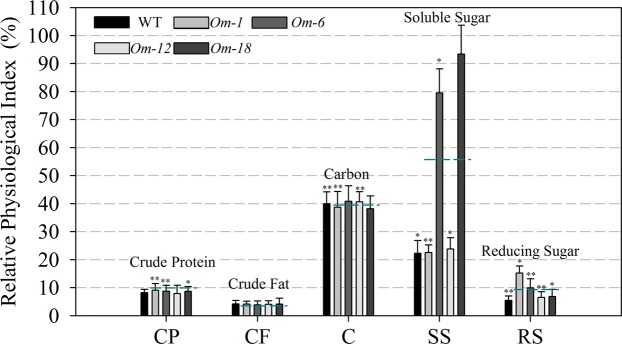
Physiological and biochemical characteristics of endosperm in *ZmMADSla-*overexpressing maize lines and wild-type. CP, Crude protein content; CF, Crude fat content; C, Carbon content; SS, Reducing sugar content; RS, Soluble sugar content; WT, wild-type plants (‘ZZC01’). *Om-1*, *Om-6*, *Om-12*, and *Om-18* are four *ZmMADSla*-overexpressing lines. The dashed blue lines indicate the average values in the overexpressing lines. The asterisks indicate that the correlation coefficients were highly significantly different (*P < 0.05, **P < 0.01).Values represent the mean ± SD of triplicates.

To understand the effects of overexpressing *ZmMADS1a* on starch synthesis, we analyzed the starch content and related biochemical indicators in T_3_-generation transgenic maize seeds. The total starch, amylose, and amylopectin contents in the four *ZmMADS1a*-overexpressing maize lines were increased (Fig. 6a–c), and the expression level of the four lines were increased (Fig. S5). In these transgenic lines, the increases in total starch and amylopectin contents were 3.26% and 5.08% respectively, while the relative increase in the amylose content was considerably higher than wild-type, up to 11.42%. Comparing the physiological and biochemical characteristics of maize endosperm in the *ZmMADSla*-overexpressing lines with wild-type, we found that the contents of soluble sugars and reducing sugars were significantly increased, but that the crude protein, crude fat, and carbon contents were not significantly different than in the control line (Fig. 7, Table S6). We also compared the rice *ZmMADSla*-overexpressing T_4_-generation seeds with the non-transgenic control. The results showed that the total starch and amylose contents followed similar trends in maize and rice, especially for amylose content, which had a maximum increase of 26.62% in the transgenic rice lines *Or-8* and *Or-12* (Figs 6 and S6). We then examined the maize seeds under transmitted light, which showed that the shaded areas of the kernels from the *ZmMADS1a*-overexpressing lines were slightly larger than in wild-type seeds (Fig. 6D). All of the above results indicate that overexpressing the *ZmMADS1a* gene can affect starch (especially amylose) and sugar contents in the endosperm.

## Discussion

ZmMADS1a, a typical MADS-box transcription factor, belongs to the MIKC^c^-subgroup of the Type II MADS-box proteins (Fig. 1I). MADS-box TF genes are especially prominent on almost all levels of the gene regulatory networks that control reproductive development in flowering plants^22,23^. In recent years, research into seed and embryo development has become increasingly focused on MADS-box transcription factors in species such as rice (*Oryza sativa* L)^26^, soybean (*Glycine max* (Linn.) Merr.)^27^, tomato (*Lycopersicum esculentum*)^28^, *Arabidopsis thaliana*^8^, and strawberry (*Fragaria* × *ananassa* Duch.)^29^.

In our study, we found that *ZmMAD1a* is expressed at a relatively high level in the maize flower (tassel and silk) and endosperm (Fig. 3A). This expression pattern is similar to that found in other plants in which seed or fruit development is regulated by MADS-box genes, which are mainly expressed in the reproductive organs. *OsMADS6*, which is important for ovule development, was highly expressed in developing seeds and flowers, but was not detected in roots, leaves, and suspension cells^30^. In tomato, the genes *TAG1*, *TAGL1*, *TAGL2*, and *TAGL11*, which are related to fruit development, were only expressed in flowers and fruits^31^, and the seed growth genes, *SHP1* and *SHP2*, are expressed mainly in the carpels and control pod shattering in Arabidopsis^32^. The level of expression of *ZmMAD1a* in the endosperm increases with the increase in days after pollination until 18-DAP, after which it declines by >10-fold in 20-DAP endosperm (Fig. 3B). Similar phenomena have been observed in other species; in rice, *OsMADS6* is expressed abundantly in the ovule during meiosis, and expression in the endosperm peaks at 10 DAP and then declines slightly at 20 DAP^30^. In soybean, *GmAGL15* is expressed in the seeds during development; its expression level peaked initially at 15 d after flowering in the young embryo, reached the highest level at 30 DAP, then dropped rapidly^27^. Transcription of the tomato *SlMADS1* gene occurs mainly in sepals and green fruits, and the mRNA levels decrease significantly with fruit ripening^3^.

We found that overexpressing the *ZmMAD1a* gene had no significant effects on maize or rice traits (Fig. S2). Phylogenetic analysis also showed that ZmMADS1a groups closely with ZAG2 (*zag2*), ZmMADS1, and other proteins that function in the regulation of flower and seed development (Fig. 1III). Our results show that ZmMAD1a affects the physiological and biochemical characteristics in the endosperm of maize and rice seeds. Overexpression of *ZmMADS1a* led to altered expression of starch biosynthesis genes (Figs 4 and S1) and changes in starch composition and structure in the endosperm (Figs 5, 6, S4, S6). The expression levels of *AGPL1* (*sh2*) and *AGPS1* (*bt2*) were increased, but *AGPL2* was generally down-regulated during development of maize endosperm (Fig. 4). These genes encode subunits of the AGPase enzyme, which controls the rate-limiting step in starch production and is regulated by allosteric effectors, and can affect the total starch content^33^. *AGPL1* (*sh2*) and *AGPS1* (*bt2*) exhibited significantly higher expression levels than *AGPL2*, which led to increases in the total starch content (Figs 6A, S6B). *GBSSI* is primarily responsible for amylose biosynthesis^34^, and *GBSSII* does not show significant activity in endosperm, but may be essential in other non-storage tissues^35^. Our results indicate that the expression of *GBSSI* and *GBSSII* show different trends during endosperm development. The *GBSSI* (*wx*) gene was significantly up-regulated and *GBSSII* was down-regulated during maize endosperm development (Fig. 4). The increased level of *GBSSI* expression in response to overexpression of *ZmMADS1a* leads to an increase in amylose content (Fig. 6B). SS genes are exclusively involved in amylopectin biosynthesis, and their distribution within the plastid between the stroma and starch granules varies between species, tissue, and developmental stages^36^. The expression of the *SSI* and *SSII* genes showed very different patterns in seeds of maize and rice *ZmMADS1a*-overexpressing lines. *SSI* is the major SS in cereal endosperm, but expression of the *SSI* gene was not drastically changed in seeds of *ZmMADS1a*-overexpressing vs. wild-type seeds (Figs 4, S1). *SSIIa* is mainly responsible for the synthesis of branched starch of medium chain length^37^, and *SSIIIa* can control the branched structure through its interaction with DBE^38^. Expression of *SSIIa* was significantly up-regulated at 15 and 20 DAP, while the *SSIIIa* gene was up-regulated at 15 DAP compared to the control (Fig. 4); therefore, the resulting amylopectin contents were changed only slightly (Fig. 6C). Expression of the *BEI* gene was down-regulated, particularly at 15 and 20 DAP. *BEIIa* was down-regulated at 20 and 25 DAP, and *BEIIb* showed significant up-regulation from 10 to 25 DAP in maize endosperm (Fig. 4). Isoamylases can play vital roles in starch granule initiation^33^. The relative expression level of *ISA* can change starch granule structure in potato, and also in Arabidopsis mutants^39^. *PUL* mainly plays a role in starch degradation, but is also involved in starch synthesis, and mutations in this enzyme affect starch content and structure^40,41^. *ISA1* (*su1*) expression was sharply up-regulated at 15 DAP, and *PUL* was down-regulated at 20–25 DAP compared to wild-type (Fig. 4). Changes in the expression levels of these genes can partially account for the observed reduction in the size of starch granules in the endosperm of *ZmMADS1a*-overexpressing transgenic maize lines (Fig. 5). The changes in the *ZmMADS1a*-overexpressing lines were not caused by a single starch gene, but rather by multiple genes which may interact and affect the expression of one another at different stages of seed development. These results indicate that *ZmMADS1a* mainly functions at the mid-late grain-filling period of kernel development in maize, and that it positively regulates the expression of endosperm starch biosynthesis genes.

Starch granules consist of ordered crystalline and disordered amorphous areas, and are composed of amylose and amylopectin in different proportions. Crystalline and amorphous structures are mainly the information reflected in the C1 signal zone in the ^13^C{^1^H}CP/MAS spectra^41^.The peak locations in our study were consistent with the results of previous studies^42^. The signal at 102.4 ppm is associated with the amorphous part of the granule. Signals at 100.3 and 101.2 ppm are associated with the crystalline structure. The signal at 82 ppm was attributed to C4 of the V-type starch, which is amylose in a single helix conformation, and the 82 ppm signal in the C4 region provides information on the amorphous components of the starch^43^. The chemical shifts are shifted to the left by ~0.5 ppm and the peak area of C4 increased noticeably in the *ZmMADS1a*-overexpressing lines (Fig. S4). Overexpression of *ZmMADS1a* had a significant impact on the disordered structures in the starch granules.

The *ZmMADS1a* gene is involved in the expression of multiple genes associated with enhanced sugar contents (reducing and soluble sugars) (Fig. 7), and the *ZmMADS1a*-overexpressing transgenic plants exhibited better growth under drought stress conditions. We provide candidate regulators and show that *ZmMADS1a* has a similar adjustment mechanism for starch biosynthesis in maize and rice grain and can enhance the expression of most starch biosynthesis genes, especially the expression of *GBSSIa* (*wx*), resulting in increased amylose content in mature seeds. The results presented here will further our understanding of the *ZmMADS1a* regulatory mechanism in endosperm starch synthesis in maize.

## Materials and Methods

### Plant material and growth conditions

Maize plants for laboratory analyses were grown in a greenhouse at a temperature of 28 ± 2 °C and a photoperiod of 14 hours light/10 hours dark. Rice seedlings were grown at 30 °C under a 16/8-h light/dark cycle in controlled environment chambers and were then transferred to the field and grown under normal rice cultivation conditions at the Experimental Station of Anhui Agricultural University, Hefei, China. Seeds were sown in a seed bed in mid-May and transplanted to the field in mid-June.

### Isolation of the *ZmMADS1a* cDNA and DNA Sequence Analysis

The *ZmMADS1a* gene sequence was obtained from MaizeSequence (http://www.maizesequence.org/index.html, GRMZM2G160687_T01, 1473 bp). Protein sequence analysis was performed using Pfam (http://pfam.janelia.org/search/sequence) and EXPASY (http://web.expasy.org/compute_pi/). Amino acid sequences were predicted using the DNAMAN software package (Lynnon Biosoft). The full-length cDNA of *ZmMADS1a* was amplified by RT-PCR from RNA extracted from seeds of maize genotype B73 16 days after pollination with primers *MA*-F and *MA*-R.

### Generation of DNA constructs and transformation

The amplified *ZmMADS1a* cDNA from the maize B73 inbred line was digested with *KpnI* and *SmaI* (primers: mMA-F-1 and mMA-R-1, Table S1) and then inserted into the p1 vector, which is a modied version of pCAMBIA1301^44^, resulting in a vector called p1-*ZmMADS1a*. These two clones were digested with *EcoRI* and *SmaI* and inserted into the binary vector pCAMBIA1301 under the control of the *Cauliflower mosaic virus* 35S (CaMV-35S) promoter, and the NOS terminator was inserted between the *SphI* and *HindIII* sites.

The pZZ00005 vector (China National Seed Group Co., Ltd) was used for overexpression analysis in maize. The 35S*::ZmMADS1a::NOS* gene cassette was amplifed from p1-*ZmMADS1a* using primers mMA-F-2 and mMA-R-2 (Table S1) carrying terminal *HindIII* and *PmeI* sites and inserted into the pZZ00005 vector, giving p2-*ZmMADS1a*. *Zea mays* ‘ZZC01’ (China National Seed Group Co., Ltd) was the maize line used for transformation.

The p1-*ZmMADS1a* vector was introduced into the *Agrobacterium* strain EHA105, and transformed into the rice (*Oryza sativa* L. subsp. *japonica*) cultivar ‘Zhonghua 11’ as described previously^45^. Using hygromycin selection, 18 independent T_0_ transgenic lines were obtained; expression of the *ZmMADS1a* transgene was confirmed in these lines by RT-PCR, and homozygous T_3_ transgenic lines were obtained for further analysis.

The p2-*ZmMADS1a* vector was also introduced into the *Agrobacterium* strain EHA105. Timentin (100 mg/L) and bialaphos (5 mg/L) were added to the selective medium. Using a protein test strip for bialaphos selection, 20 independent T_0_ transgenic lines were obtained and the homozygous T_3_ transgenic lines were obtained for further analysis.

### Gene expression analyses

The relative expression of each endosperm gene was analyzed in eight different maize tissues by qRT-PCR, and the α-tubulin gene (GRMZM2G152466_T01) was used as an internal control for normalization of gene expression levels. PCR primer pairs were designed with Primer Express 3.0 using the B73 genome sequence as a reference (MaizeGDB release 5b.60; http://www.maizegdb.org/). To study the expression pattern of *ZmMADS1a*, 16 different tissues, including stage-3 root, stage-3 stem, stage-3 leaf, tassel, cornsilk, stegophyl, 12 DAP_endosperm, 12 DAP_embryos, 14 DAP_endosperm, 14 DAP_embryos, 16 DAP_endosperm, 16 DAP_embryos, 18 DAP_endosperm, 18 DAP_embryos, 20 DAP_endosperm, and 20 DAP_embryos were collected (DAP = days after pollination).

To analyze the expression patterns of starch synthesis genes in seeds of transgenic and wild-type plants, developing seeds were harvested from maize (5 DAP, 10 DAP, 15 DAP, 20 DAP, and 25 DAP) and rice (3 DAP, 6 DAP, 10 DAP, 15 DAP, 20 DAP, and 25 DAP). All samples were frozen immediately in liquid nitrogen and stored at −80 °C prior to use. Total RNA was isolated using the E.Z.N.A. MagSi Plant RNA Kit (Omega Biotek), and was treated with DNaseI to remove contaminating genomic DNA. All PCR primers used in this study are given in Tables S2, S3. First-strand cDNAs were synthesized using the PrimeScript™ RT reagent Kit with gDNA Eraser (Perfect Real Time; Takara). Expression levels were calculated using the 2^−ΔΔCT^ method of Livak and Schmittgen (2001)^46^ and statistically analyzed as described by Liu *et al*.^47^.

### Subcellular localization

Three fusion vectors were constructed to study the localization of ZmMADS1a. The full-length cDNA sequence of *ZmMADS1a* without the terminator codon (TGA) was amplified from B73 RNA. The identity of the gene fragment was confirmed by DNA sequencing and was then inserted between the *HindIII* and *BamHI* sites of the pEZS-NL vector, which contains the enhanced green fluorescent protein (eGFP) reporter gene, to generate a fusion construct under the control of the CaMV 35S promoter, namely pEZS-*MS*-eGFP (the primer names were MA-SL-F and *MA*-SL-R, Table S1). The localization vector was transferred into protoplasts of Arabidopsis and maize by electroporation. The leaves of Arabidopsis were selected for 4 weeks after the leaves were not smoked about 90 pieces and the leaves of maize were selected 2 weeks of growth. The two kinds of leaves were cut into 1 mm wide strips.

### Grain trait measurements

More than three randomly chosen, fully filled grains from each line or strain were aligned length-wise along a vernier caliper to measure seed length, after which they were rearranged to measure grain width and thickness^48^. The 1,000-grain weight was determined by weighing 10 replicates of 100-grain samples independently on an electronic balance.

### Analysis of starch properties

Harvested paddy seeds were air-dried and stored at room temperature. Embryos and pericarps were removed before the experiment, and the endosperms were ground to a powder using a grinding mill. The starch and amylose contents were measured using starch assay kits following the manufacturer’s instructions (K-TSTA and K-AMYL; Megazyme). To determine the amylose content, the powder was soaked for 48 h in 0.4% NaOH (powder:NaOH = 1:3) at room temperature, washed several times with distilled water until no slimy liquid remained, and drained. To determine the amount of soluble sugars, 50 mg of endosperm powder was washed twice in 80% (v/v) ethanol at 80 °C for 40 min and assayed using anthrone reagent^49^.

### Observation of endosperm starch granules

Seeds were dried completely under low pressure, cut across the short axis, and the surface was sputter coated with gold prior to observation with a scanning electron microscopy. To analyze the structure of starch granules, solid-state ^13^C CP/MAS NMR spectra was used. About 0.3 mg of starch samples were packed into a 4-mm diameter, cylindrical, partially stabilized zirconium oxide (PSZ) rotor (6 kHz, 54.7°) with a Kelf and cap. The experiments were conducted at a ^13^C frequency of 75.46 MHz on a Bruker MSL-300 spectrometer^43,50,51^.

## Supplementary information

Supplementary Figures and Supplementary Tables
