# Supplementary material for: Functional analysis of ZmMADS1a reveals its role in regulating starch biosynthesis in maize endosperm
Source: Sci Rep. 2019 Mar 1;9:3253. doi: 10.1038/s41598-019-39612-5 (PMC6397188; doi:10.1038/s41598-019-39612-5)
Supplement: Supplementary file 1 — Supplementary Figures and Supplementary Tables [file 41598_2019_39612_MOESM1_ESM.docx]

**Functional analysis of *ZmMADS1*a reveals its role in regulating starch biosynthesis in maize endosperm**

Qing Dong^1,2,#^, Fang Wang^1,^^#^, Jingjing Kong^2, #^, Qianqian Xu^2^, Tingchun Li^1^, Long Chen^2^, Hongjian Chen^1^, Haiyang Jiang^2^, Cheng Li^1,^* & Beijiu Cheng^2,^*

^#^Authors who contributed equally to this work

*Corresponding author: Cheng Li; Beijiu Cheng

Email: [beijiucheng@ahau.edu.cn](mailto:beijiucheng@ahau.edu.cn); ahnkyrsc@163.com

^1^Maize Research Center, Anhui Academy of Agricultural Sciences, Hefei, 230031, China.

^2^National Engineering Laboratory of Crop Stress Resistence, Anhui Agricultural University, Hefei, 230036, China.

**Supplementary Figures**

**
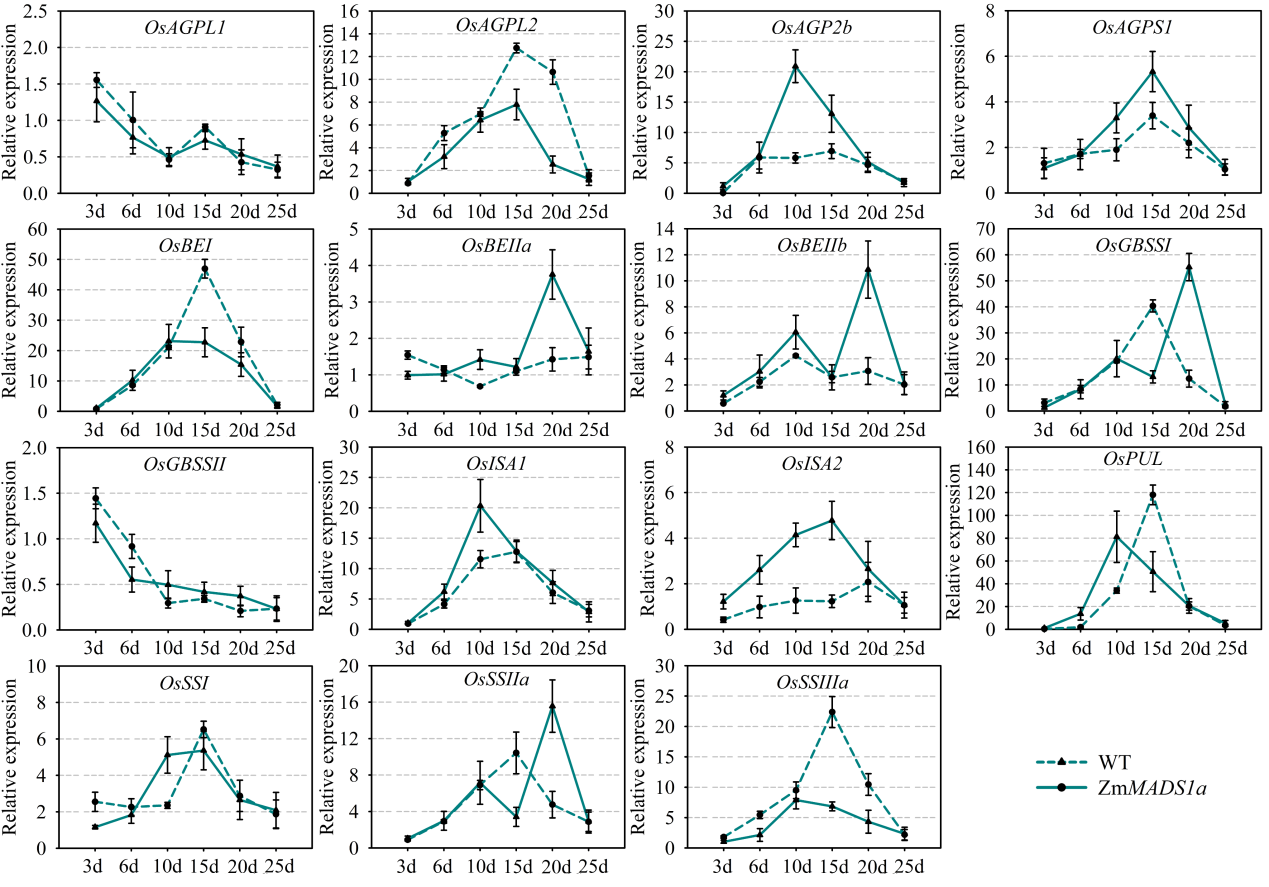
**

**Fig. S1** Relative expression levels of 15 rice starch biosynthesis genes during seed development in ‘Zhonghua 11’ (wild-type) and the *ZmMADSla-*overexpressing lines. The expression of each gene in the 3-DAP endosperm of ‘Zhonghua 11’ was used as the control. Values represent the mean±SD of three replicates.

**
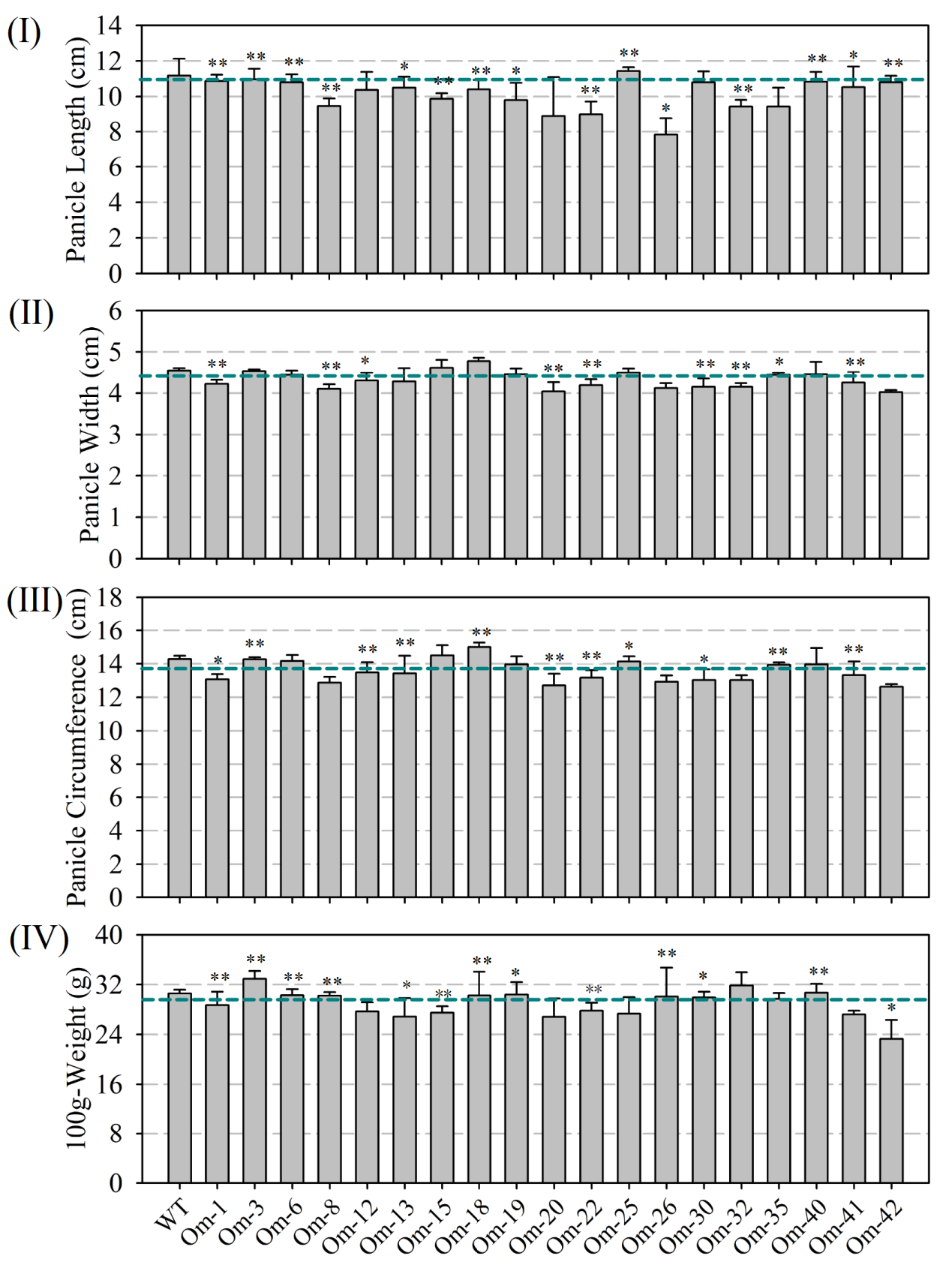
**

**Fig. S2** Seed weight and panicle characters in the wild-type and 19 *ZmMADS1a-*overexpressing maize lines. **(I)** Panicle length; **(II)** Panicle width; (**III**) Panicle circumference; **(IV)** 100-seed weight; *Om-*, *ZmMADS1a*-overexpressing transgenic maize lines; WT, wild-type plants (‘ZZC01’). The dashed blue line indicates the average value for the 19 overexpressing lines. Values represent the mean±SD of three replicates. The asterisks indicate that the correlation coefficients were highly significantly different (*P<0.05, **P< 0.01).

**
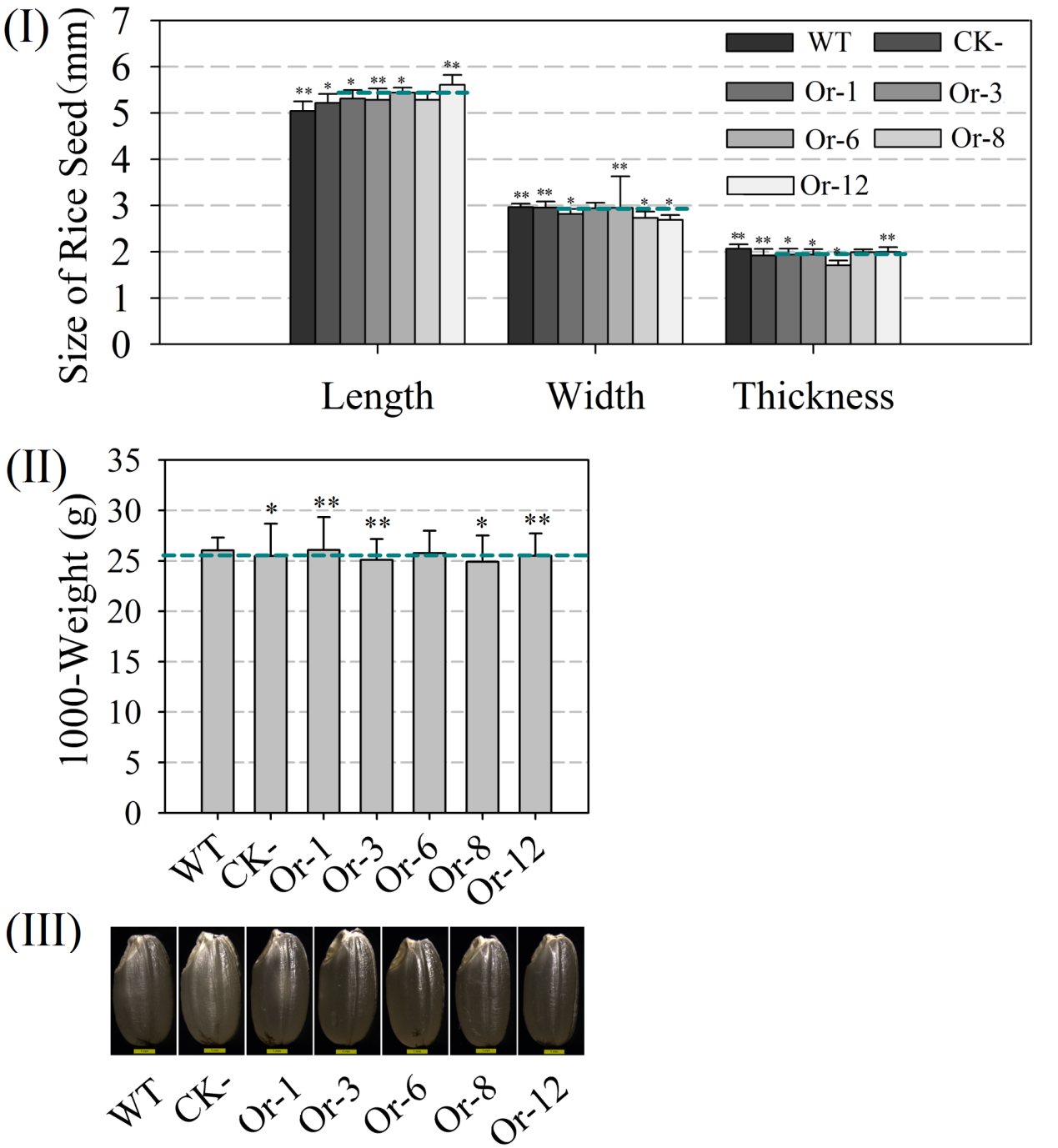
**

**Fig. S3** Seed size and weight in the wild-type and five *ZmMADS1a-*overexpressing rice lines. (**I**) Size of rice seed including length, width and thickness. (**II**) 1000-seed weight. Or**-**1, -3, -6, -8, and -12 are the *ZmMADS1a*-overexpressing lines; WT:, wild-type plants (‘Zhonghua 11’); CK-, p1301a vector transgenic lines; Values represent the mean ±SD of three replicates. The dashed blue lines indicate the average values for the five overexpressing lines. The asterisks indicate that the correlation coefficients were highly significantly different (*P<0.05, **P< 0.01).

**
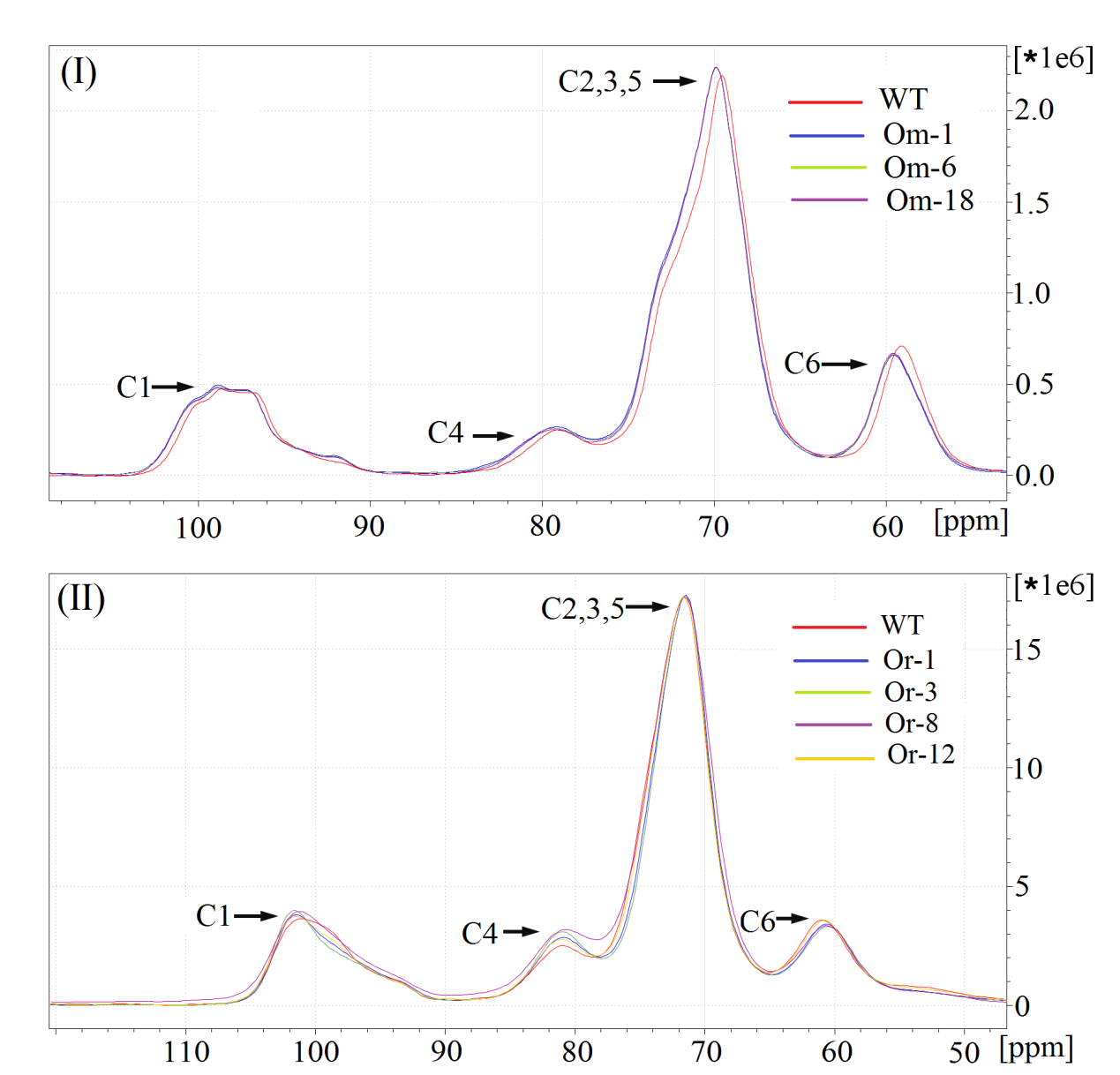
**

**Fig. S4** Analysis of starch granule structure by solid-state ^13^C CP/MAS NMR spectra. (**I**) NMR analysis in maize; (**II**) NMR analysis in rice; Om-1, -6, and -18 are three *ZmMADS1a*-overexpressing maize lines; Or-1, -3, -8, and -12 are four *ZmMADS1a*-overexpressing rice lines; (I, WT), wild-type maize plants (‘ZZC01’); (II, WT), wild-type rice plants (‘Zhonghua 11’); C, Carbon atoms in glucose units.

**
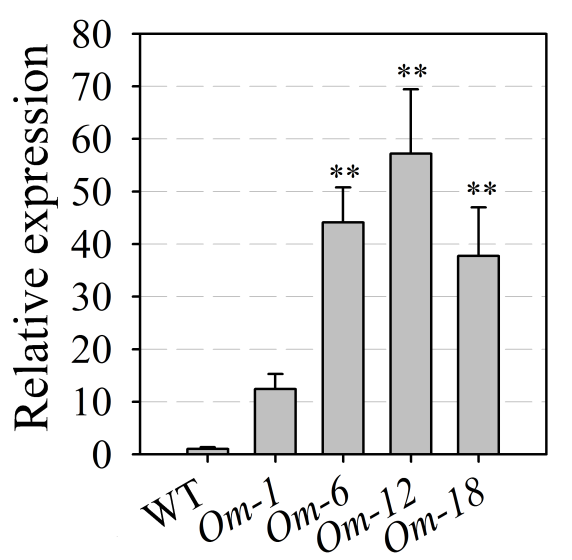
**

**Fig. S5** Expression levels of *ZmMADS1a* in the four transgenic lines. *Om-1*, *Om-6*, *Om-12*, and *Om-18* are four overexpressing lines of *ZmMADSla* seeds; WT, wild-type plants (‘ZZC01’). The dashed blue lines indicate the average for the overexpressing lines. The asterisks indicate that the correlation coefficients were highly significantly different (*P<0.05, **P < 0.01).Values represent the mean±SD of triplicates.

**
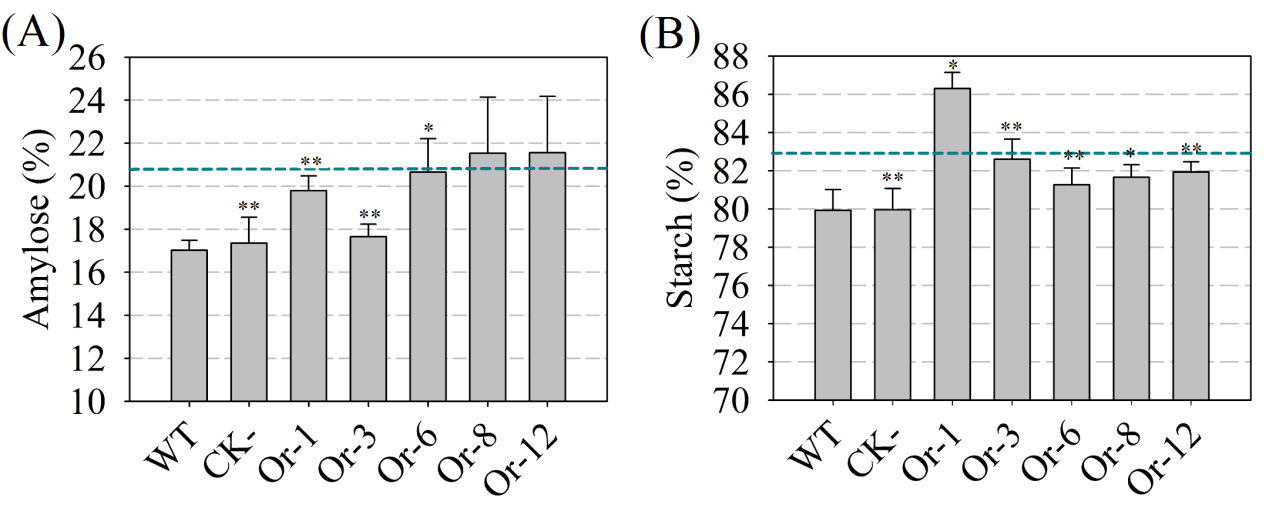
**

**Fig. S6** Comparison of the total starch and amylose contents in seeds of wild-type and five *ZmMADS1a*-overexpressing lines; **(A)** Total starch content; **(B)** Amylose content; Or-1, -3, -6, -8, and -12 are five *ZmMADS1a*-overexpressing lines of rice; WT, wild-type plants (‘Zhonghua 11’); CK**-**, transgenic lines from the p1301a control vector. The dashed blue lines show the average values for the overexpressing lines.

**Supplementary Tables**

**Table S1 Nucleotide sequences of oligonucleotide primers used in this study**

Restriction enzyme recognition sites are underlined

| **Assay** | **Primer Name** | **Sequence(5'-3')** |
| --- | --- | --- |
| **Gene Clone** | MA-F | ATGGGTAGGGGAAGGATTGA |
|  | MA-R | TTAGAACTGATGATGAGGGTTATTG |
| **Genetic Transformation** | mMA –F-1 | GGGGTACCATGGGTAGGGGAAGGATTGA |
| **Transformation** | mMA –R-1 | TCCCCCGGGTTAGAACTGATGATGAGGGTTAT |
|  | mMA –F-2 | CCCAAGCTT ATGGGTAGGGGAAGGATTGA |
|  | mMA–R-2 | AGCTTTGTTTAAA TTAGAACTGATGATGAGGGTTAT |
| **Subcellar Location** | MA-SL1-F | GGGAAGCTTATGGGTAGGGGAAGGATTGAGATC |
|  | MA-SL1-R | GGGGGATCCCGGAACTGATGATGAGGGTTATTGTCG |

**Table S2 Primers used to study gene expression in maize starch biosynthesis genes**

| **Gene name** | **Acc.no.** | **NCBI ID** | **Primer sequence (5´-3´)** | **Amplicon size (bp)** |
| --- | --- | --- | --- | --- |
| *ZmAGPS1a-1* | AF330035 | GRMZM2G068506_T01 | F:GCACAGCAGAGCCCAGATAA | 133 |
|  |  |  | R:CCATCCGGTACAGGTGATCG |  |
| *ZmAGPL1(sh2)* | JX462603 | GRMZM2G429899_T01 | F:AGCCACAAGATCACTTCGGG | 134 |
|  |  |  | R:GGTGAGGACCTGAGTTCGTG |  |
| *ZmAGPL2* | Z38111 | GRMZM2G027955_T02 | F:GACGCGATCATTTCACACGG | 164 |
|  |  |  | R:CCTCTGCCAGTAGCCTTGAG |  |
| *ZmGBSSIa (wx)* | HQ423253 | GRMZM2G024993_T01 | F:AAGACCGCTTTCTGCATCCA | 146 |
|  |  |  | R:TTGATCTTCCGGCCTTCCAC |  |
| *ZmGBSSIIa* | EF471312 | GRMZM2G008263_T01 | F:ACCGGATTCCACATGGGTTC | 193 |
|  |  |  | R:GAAGCACCTCCTCCCACTTC |  |
| *ZmSSIa* | AF036891 | GRMZM2G129451_T02 | F:ATTCGCACCCCTAACCACAG | 146 |
|  |  |  | R:CGTATTGTTCAGCGGCATGG |  |
| *ZmSSIIa (su2)* | AF019296 | GRMZM2G348551_T01 | F:TTGATGCCCCTCTTTTCCGG | 145 |
|  |  |  | R:TCAGGCAAGTCCATGTACGG |  |
| *ZmSSIIIa (du1)* | AF023159 | GRMZM2G141399_T01 | F:AGGGTCATTCGATGCGTTGT | 166 |
|  |  |  | R:CTTCACGGTGTCTGCTGCTA |  |
| *ZmBEI* | AF072724 | GRMZM2G088753_T01 | F:AATGGCCCTTGGAGGTGATG | 178 |
|  |  |  | R:CGCTTGGTCAAACGCATTCA |  |
| *ZmBEIIa( 2a)* | U65948.1 | GRMZM2G073054_T02 | F:GCGTTGGTTGTTTCAAGCCT | 182 |
|  |  |  | R:AGGTGCATATACGACGGCTG |  |
| *ZmBEIIb (ae)* | EF433557 | GRMZM2G032628_T01 | F:GCGCAACCTAAACGACCAA | 68 |
|  |  |  | R:GGTTCCGGGCTACTCATTCC |  |
| *ZmISA1 (su1)* | ZMU18908 | GRMZM2G138060_T01 | F:TGCCCTGCCATGAGTTCAAT | 174 |
|  |  |  | R:GGGCCTCCCTTACAAAAGCT |  |
| *ZmISA2(ISO2)* | EU976060 | GRMZM2G090905_T01 | F:CGCGGTTTACAGCAAGATCG | 132 |
|  |  |  | R:ATCGAGACGTGCCAGACATC |  |
| *ZmPUL (zpu1)* | AF080567 | GRMZM2G158043_T02 | F:TGGCTCACCAGTTGGCTATG | 133 |
|  |  |  | R:CCTGCATCGCTCGTCAATTG |  |
| *ZmPHOL (sh4)* | EU857640 | GRMZM2G074158_T01 | F:GAAGGTTACGGACGTGCAGA | 163 |
|  |  |  | R:TCGTCCTATCGCTGCTGAAC |  |

**Table S3. Primers used to study gene expression in rice starch biosynthesis genes**

| **Gene name** | **Acc.no.** | Primer sequence 5´-3´ | **Amplicon size (bp)** |
| --- | --- | --- | --- |
| *OsAGPL1* | D50317 | F:GGAAGACGGATGATCGAGAAAG | 140 |
|  |  | R: CACATGAGATGCACCAACGA |  |
| *OsAGPL2* | U66041 | F: AGTTCGATTCAAGACGGATAGC | 96 |
|  |  | R: CGACTTCCACAGGCAGCTTATT |  |
| *OsAGPS1* | AK073146 | F: GTGCCACTTAAAGGCACCATT | 97 |
|  |  | R: CCCACATTTCAGACACGGTTT |  |
| *OsAGPS2b* | AK103906 | F: AACAATCGAAGCGCGAGAAA | 186 |
|  |  | R: GCCTGTAGTTGGCACCCAGA |  |
| *OsBEI* | D11082 | F: TGGCCATGGAAGAGTTGGC | 191 |
|  |  | R: CAGAAGCAACTGCTCCACC |  |
| *OsBEIIa* | AB023498 | F: GCCAATGCCAGGAAGATGA | 128 |
|  |  | R: GCGCAACATAGGATGGGTTT |  |
| *OsBEIIb* | D16201 | F: ATGCTAGAGTTTGACCGC | 261 |
|  |  | R: AGTGTGATGGATCCTGCC |  |
| *OsGBSSI* | X62134 | F: AACGTGGCTGCTCCTTGAA | 218 |
|  |  | R: TTGGCAATAAGCCACACACA |  |
| *OsGBSSII* | AY069940 | F: AGGCATCGAGGGTGAGGAG | 246 |
|  |  | R: CCATCTGGCCCACATCTCTA |  |
| *OsISA1* | AB093426 | F: TGCTCAGCTACTCCTCCATCATC | 132 |
|  |  | R: AGGACCGCACAACTTCAACATA |  |
| *OsISA2* | AC132483 | F: TAGAGGTCCTCTTGGAGG | 170 |
|  |  | R: AATCAGCTTCTGAGTCACCG |  |
| *OsISA3* | AP005574 | F:ACAGCTTGAGACACTGGGTTGAG | 100 |
|  |  | R: GCATCAAGAGGACAACCATCTG |  |
| *OsPUL* | AB012915 | F: ACCTTTCTTCCATGCTGG | 202 |
|  |  | R: CAAAGGTCTGAAAGATGGG |  |
| *OsSSI* | D16202 | F: GGGCCTTCATGGATCAACC | 279 |
|  |  | R: CCGCTTCAAGCATCCTCATC |  |
| *OsSSIIa* | AF419099 | F: GCTTCCGGTTTGTGTGTTCA | 54 |
|  |  | R: CTTAATACTCCCTCAACTCCACCAT |  |
| *OsSSIIIa* | AY100469 | F: GCCTGCCCTGGACTACATTG | 334 |
|  |  | R: GCAAACATATGTACACGGTTCTGG |  |
| *OsSSIVa* | AY100470 | F: GGGAGCGGCTCAAACATAAA | 237 |
|  |  | R: CCGTGCACTGACTGCAAAAT |  |

**Table S4 . Comparison of traits between wild-type and *ZmMADS1a*-overexpressing lines in rice**

| **Trait** | **WT** | ***ZmMADS1a*** |
| --- | --- | --- |
| Plant Height (cm) | 118.32±5.67 | 117.42±7.17 |
| No. of panicles per plant | 12.58±3.86 | 12.55±3.66 |
| No. of grain per panicle | 150.82±14.45 | 156.55±18.24 |
| No. of filled grains per panicle | 150.42±13.26 | 155.08±15.21 |

**Table S5. Comparison of traits between wild-type and *ZmMADS1a-*overexpressing lines in maize**

| **Trait (cm)** | **WT** | ***ZmMADS1a*** |
| --- | --- | --- |
| Plant Height | 145.65±5.66 | 150.20±8.42 |
| Ear Length | 55.08±5.55 | 57.97±8.06 |
| Leaf Length | 62.85±6.55 | 62.2±8.25 |
| Leaf Width | 9.42±0.85 | 8.89±1.02 |

Data are presented as mean ± SD

**Table S6. Relative physiological indexes measured in wild-type and *ZmMADS1a* -overexpressing maize lines**

|  | **crude protein%** | **crude fat%** | **carbon%** | **soluble sugars%** | **reducing sugars%** |
| --- | --- | --- | --- | --- | --- |
| WT | 8.25±1.21 | 4.23±0.58 | 39.99±1.55 | 22.29±3.52 | 5.50±0.52 |
| *Om-1* | 9.13±2.38 | 4.18±1.03 | 38.71±5.67 | 22.58±2.68 | 15.27±2.49 |
| *Om-6* | 8.78±2.08 | 3.91±1.34 | 40.87±5.52 | 79.52±8.65 | 9.97±3.25 |
| *Om-12* | 7.90±3.02 | 4.11±1.25 | 40.67±3.64 | 23.82±4.05 | 6.5±2.06 |
| *Om-18* | 8.70±1.69 | 4.12±2.15 | 38.25±4.55 | 93.42±10.28 | 6.84±2.6 |

Data are presented as mean ± SD

**Table S7. Chemical shifts in the NMR spectra of endosperm starch from the *ZmMADS1a-* overexpressing maize lines and the wild-type**

| **ppm** | **C1** | **C4** | **C2、C3、C5** | **C6** |
| --- | --- | --- | --- | --- |
| WT | 100.14、98.64、96.68、91.51 | 79.11 | 69.6 | 59.16 |
| *Om-1* | 100.56、99.00、97.25、92.02 | 79.16 | 69.98 | 59.60 |
| *Om-6* | 100.56、99.00、97.25、91.74 | 79.34 | 69.98 | 59.60 |
| *Om-18* | 100.56、99.00、97.25、91.92 | 79.34 | 69.98 | 59.69 |

**Table S8. Calculation of peak areas in the NMR spectra of endosperm starch from the *ZmMADS-* overexpressing maize lines and the wild-type**

| Carbon atoms | Chemical shift (ppm) | WT(100%) | *Om-1* | *Om-6* | *Om-18* |
| --- | --- | --- | --- | --- | --- |
| C1 | 112.00-88.50 | 16.85% | 17.40% | 17.19% | 16.98% |
| C4 | 88.50-78.40 | 4.24% | 5.09% | 4.91% | 4.61% |
| C2,C3,C5 | 78.40-65.10 | 63.35% | 63.30% | 63.35% | 63.36% |
| C6 | 65.10-52.10 | 15.56% | 14.21% | 14.55% | 15.05% |

**Table S9. Calculation of peak areas in the NMR spectra of endosperm starch from the *ZmMADS1a-* overexpressing rice lines and the wild-type**

| **ppm** | **C1** | **C4** | **C2、C3、C5** | **C6** |
| --- | --- | --- | --- | --- |
| WT | 100.98 | 80.90 | 71.62 | 60.99 |
| *Or-1* | 101.40 | 80.90 | 71.60 | 60.60 |
| *Or-3* | 101.50 | 80.90 | 71.60 | 60.60 |
| *Or-8* | 101.50 | 80.90 | 71.60 | 60.60 |
| *Or-12* | 101.70 | 80.90 | 71.60 | 60.60 |

**Table S10. Chemical shifts in the NMR spectra of endosperm starch from the *ZmMADS1a -* overexpressing rice lines and the wild-type**

| Carbon atoms | Chemical shift (ppm) | WT (100%) | *Or-1* | *Or-3* | *Or-8* | *Or-12* |
| --- | --- | --- | --- | --- | --- | --- |
| C1 | 112-88.5 | 17.19% | 18.57% | 17.26% | 17.30% | 17.28% |
| C4 | 88.5-78.4 | 7.93% | 10.00% | 8.62% | 8.86% | 9.49% |
| C2,C3,C5 | 78.4-65.1 | 60.92% | 59.26% | 60.26% | 60.54% | 59.95% |
| C6 | 65.1-52.1 | 13.96% | 12.17% | 13.85% | 13.30% | 13.28% |
